# Apamin-Conjugated Alendronate Sodium Nanocomplex for Management of Pancreatic Cancer

**DOI:** 10.3390/ph14080729

**Published:** 2021-07-27

**Authors:** Nabil A. Alhakamy, Osama A. A. Ahmed, Usama A. Fahmy, Shadab Md

**Affiliations:** 1 Department of Pharmaceutics, Faculty of Pharmacy, King Abdulaziz University, Jeddah 21589, Saudi Arabia; nalhakamy@kau.edu.sa (N.A.A.); oaahmed@kau.edu.sa (O.A.A.A.); 2 Center of Excellence for Drug Research and Pharmaceutical Industries, Faculty of Pharmacy, King Abdulaziz University, Jeddah 21589, Saudi Arabia; 3 Mohamed Saeed Tamer Chair for Pharmaceutical Industries, Faculty of Pharmacy, King Abdulaziz University, Jeddah 21589, Saudi Arabia

**Keywords:** apamin, apoptosis, alendronate sodium, nanocomplex, pancreatic cancer, peptide

## Abstract

Pancreatic cancer has a low survival rate and has limited therapeutic options due to the peculiarity of the tumor tissue. Cancer nanotechnology provides several opportunities to resolve such difficulties as a result of the high surface-to-volume ratio of nanostructures. Peptide-drug nanocomplexes have proved to have immense potential in anticancer activity against pancreatic cancer cells. Thus, in the present study apamin (APA) and alendronate sodium (ALS) were combined to form nanocomplexes (APA-ALS-NC) against pancreatic cancer cells. Optimization of ALS, incubation time, and sonication time in terms of particle size of the nanocomplex was carried out. The optimized formulation was evaluated for anticancer activities in pancreatic cancer cells (PANC-1 cells). A Box-Behnken design using ALS, incubation time, and sonication time as independent factors and particle size as the response was chosen to optimize the APA-ALS-NC formulation. The optimized APA-ALS-NC had a particle size of 161.52 ± 8.4 nm. The evaluation of APA-ALS-NC in PANC-1 cells was carried out using various in vitro tests. The IC_50_ values were determined by MTT assay and found to be 37.6 ± 1.65, 13.4 ± 0.59, and 1.01 ± 0.04 µg/mL for ALS, APA, and APA-ALS-NC, respectively. The higher cytotoxicity activity of APA-ALS-NC was confirmed from the higher percentage of cells in the necrosis phase (apoptosis study) and the G2-M phase (cell cycle study) compared to that of ALS and APA. While the loss of mitochondrial membrane potential was less for APA-ALS-NC, the levels of IL-1β, TNF-α, caspase-3, ROS, IL-6, and NF-kB showed that APA-ALS-NC can significantly enhance apoptosis and cytotoxicity in PANC-1 cells. Moreover, Bax (10.87 ± 1.36), Bcl-2 (0.27 ± 0.02), and p53 (9.16 ± 1.22) gene expressions confirmed that APA-ALS-NC had a significant apoptotic effect compared to ALS and APA. In summary, the APA-ALS-NC had a more significant cytotoxic effect than ALS and APA. The results of the present study are promising for further evaluation in pre-clinical and clinical trials for arriving at a successful therapeutic strategy against pancreatic cancer.

## 1. Introduction

Pancreatic cancer has emerged to be one of the leading causes of cancer-associated mortality and it has a distressing 5-year survival period [1]. The availability of few successful therapeutic approaches and its inoperability makes pancreatic cancer more serious than other cancers [2]. The major hurdle in drug therapeutics in pancreatic cancer is the restricted blood supply. This poses major concerns in drug targeting to pancreatic cancer cells [3]. Pancreatic cancer cells, however, are able to adjust themselves to hypoxia and they depend less on blood vessels. Moreover, the adjacent cancer stem cells also cause hindrance to chemotherapy. These worsen the scenario and reduce the probability of successful therapeutics. Thus, systemic delivery of chemotherapeutic agents cannot be effective and some advanced therapeutic approaches are needed. Targeting the metabolic pathways and the presence of an acidic microenvironment can be considered as opportunities for the development of advanced drug delivery systems. Surgery, radiation, and chemotherapy are the present treatment strategies for pancreatic cancer [4].

Targeting drugs to pancreatic cancer could offer higher efficacy and reduced toxicity. Several approaches can be tried to target pancreatic cancer [5]. Targeting transmembrane receptor proteins, such as EGFR, insulin-like growth factor 1 receptor (IGFR), and vascular endothelial growth factor (VEGF) receptor, is one of these [5,6]. Overexpression of such receptors in pancreatic cancer is an advantage for drug targeting. Targeting the RAS signaling pathway is yet another approach but the compensatory mechanisms and toxicities are some limitations [7]. Meanwhile, targeting the acidic tumor microenvironment is a widely reported strategy for tumor-targeted delivery. This approach has been tried for pancreatic ductal adenocarcinoma [8]. Targeting cancer stem cells and DNA damage repair systems are two other promising approaches still under evaluation [5].

Peptide-mediated pancreatic-cancer targeting and imaging have emerged significantly in recent times [9,10]. Apamin (APA), a peptide found in bee venom (apitoxin), has been used in targeted drug delivery [11]. APA has a plethora of pharmacological actions which could be tailored for therapeutic applications [12]. In addition, the cytotoxic activity of bee venom components is also reported [13]. Meanwhile, alendronate sodium (ALS) is a bisphosphonate that can be used as both a therapeutic agent and targeting moiety. The cytotoxicity of ALS could be utilized successfully [14,15]. ALS is particularly useful for bone-targeted delivery of drugs [16]. Importantly, bisphosphonates have demonstrated an anti-angiogenic effect and induction of apoptosis in several cancer cells in vitro [17]. Furthermore, bisphosphonates are considered potential adjutants for tumors in the digestive system [18]. Among the bisphosphonates, nitrogen-containing bisphosphonates have better anti-tumor effects by inhibition of protein prenylation and adversely affecting cell function and survival [19]. Meanwhile, among the nitrogen-containing bisphosphonates, ALS alone has proven cofilin downregulation activity [20]. Downregulation of overexpressed cofilin in pancreatic cancer cells has been established to be a promising strategy [21,22]. In addition, ALS has sensitization action in pancreatic cells resulting in cell death [23]. Thus, ALS was chosen in the present study.

Cancer nanotechnology provides several opportunities to overcome present hurdles in cancer chemotherapeutics. The high surface-to-volume ratio of nanostructures provides several advantages as a drug carrier. Nanoparticles, nanotubes, nanovesicles, nanocapsules, nanoemulsions, nanodots, and nanowires are some of the nanostructures studied for cancer therapeutics and imaging [24]. Meanwhile, drug nanocomplexes can be considered as a special system of nanocarrier wherein the drug is conjugated or attached to a carrier part. The carrier molecule can be polymer, lipid, peptide, or even DNA [25,26,27,28]. Interestingly, peptide-drug nanocomplexes have been shown to have significant anticancer activity including against pancreatic cancer cells [29]. Nevertheless, several parameters determine the efficacy and success of the drug nanocomplexes, and optimization of these formulation parameters is required for the success of this therapeutic strategy against pancreatic cancer.

The application of statistical techniques through the design of experiments has been widely used for the optimization of pharmaceutical processes and formulations. This significantly reduces the time and effort in reaching an optimum formula with satisfactory and reliable performance [30]. In the case of nanocomplexes, optimization of particle size is very important as it has a direct relation to the tumor-cell uptake and targeting efficacies [31]. Therefore, the parameters that can influence the particle size significantly need to be optimized.

The present study aimed to formulate nanocomplexes using APA and ALS (APA-ALS-NC) against pancreatic cancer cells. Optimization of ALS, incubation time, and sonication time in terms of particle size of the nanocomplex was carried out. The optimized formulation was evaluated for anticancer activities in pancreatic cancer cells (PANC-1 cells).

## 2. Results and Discussion

### 2.1. Formulation and Optimization of APA-ALS-NC

Hydrophobic ion-pairing has been widely tried for ALS for the preparation of nanocarriers. The process involves the interaction of charged hydrophilic molecules (ALS) and oppositely charged molecules with hydrophobic groups (peptides and proteins). The resultant complex will precipitate in aqueous media as a nanostructure [32,33]. Such a hydrophobic ion-pairing complex formation can occur between ALS and APA resulting in the formation of APA-ALS-NC. Here, negatively charged alendronate can attach to positively charged groups of strongly basic APA [33,34]. The formulation and optimization of APA-ALS-NC were carried out using a three-factor, three-level Box-Behnken design (BBD). The responses obtained for various APA-ALS-NC formulation trials are presented in Table 1. The design predicted values were found to be in good agreement with the observed values.

The analysis of variance (ANOVA) data of the model is provided in Table 2. The *p*-values confirmed that ALS (Factor A), incubation time (Factor B), and sonication time (Factor C) had a significant influence on the particle size of APA-ALS-NC formulations. In addition, the interaction terms AA and AC were also found to significantly influence the particle size. The R-squared value (99.6052%) and the adjusted R-squared values (98.8947%) were observed for the design data.

The polynomial equation suggested by the software for PS is provided in Equation (1). The regression coefficients for the independent factors implied that all these selected factors influenced the particle size. The value of the regression coefficient was highest for ALS (38.1908) compared to that for incubation time (0.23559), and sonication time (11.9826). Thus, the influence of the independent factors on particle size was in the order ALS > incubation time > sonication time.

(1) $$\begin{array}{c} \mathrm{Size}=119.695+38.1908A+0.23559B+11.9826C-0.940329A^{2}+0.00138889\mathrm{AB}-1.63889\mathrm{AC}+0.00216146B^{2}+\\ 0.071875\mathrm{BC}-2.13542C^{2} \end{array}$$

**Figure 1:**
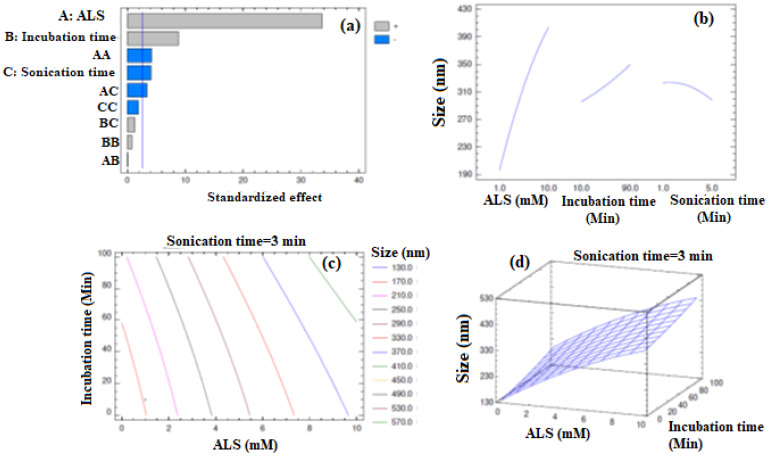
Design plots obtained for particle size of APA-ALS-NC formulations (**a**) standardized Pareto chart, (**b**) main effects plot, (**c**) contour plot, and (**d**) response surface plot.

The Pareto chart (Figure 1a) indicated significant effects of ALS, incubation time, and sonication time on particle size. The chart showed positive effects for ALS and incubation time and negative effects for sonication time. Thus, higher levels of the factors ALS and incubation time increase the particle size whereas higher levels of sonication time decrease it. These observations were in consensus with the inferences obtained from Equation (1). This behavior was confirmed from the main effects plot too (Figure 1b). The increase in particle size of the drug-lipid conjugate on increasing the drug level has been demonstrated earlier [35]. Such an effect can be also anticipated in the case of APA-ALS-NC. In the case of preparation of APA-ALS-NC, incubation time is similar to reaction time offered for the attachment of ALS (drug) to APA. Increasing reaction time increases the conjugation process, i.e., the attachment of drug [36]. Thus, increasing incubation time increases the chance for attachment of more drug molecules and subsequent increase in particle size. Therefore, an increase in the incubation time can increase the particle size. Meanwhile, the reduction of nanoparticle size on increasing the sonication time has already been demonstrated [37]. Thus, the observation of the present study can be justified. Meanwhile, the statistically significant interaction effect of AA and AC on particle size was also confirmed. Further, the interaction effects of AA and AC were found to be negative with decreasing the particle size. The iso-value curves in the contour plot were more dependent on the ALS confirming the observations of the main effects plot (Figure 1c). The significant elevation of the response surface (Figure 1d) on increasing the levels of ALS was also seen.

**Figure 2:**
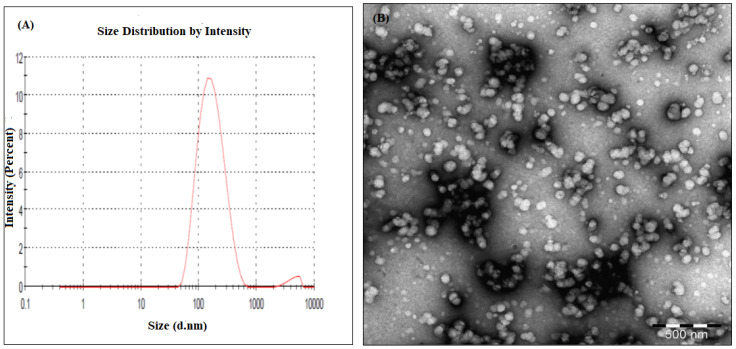
Particle size analysis using zetasizer (**A**) and TEM (**B**).

The optimum formula of APA-ALS-NC suggested by the software is shown in Table 3. The particle size, size distribution, and zeta potential for the optimum APA-ALS-NC formula were 161.52 ± 8.4 nm, 0.234 ± 0.01, and 29.67 ± 1.90 mV, respectively (Figure 2A). The actual particle size of optimized formulation determined by TEM was less than 200 nm (Figure 2B). The size distribution and zeta potential value indicate the homogenous size distribution and better stability of formulation.

### 2.2. In Vitro Cell Line Studies of APA-ALS-NC in Pancreatic Cancer Cells (PANC-1 Cells)

#### 2.2.1. IC_50_ Determination Using MTT Assay

The MTT assay was carried out in PANC-1 cells to determine the IC_50_ value. The IC_50_ values were 37.6 ± 1.65, 13.4 ± 0.59, and 1.01 ± 0.04 µg/mL for ALS, APA, and APA-ALS-NC, respectively. Thus, the nanocomplex was found to be significantly more cytotoxic (*p*-value < 0.05) than ALS and APA individually.

#### 2.2.2. Apoptotic Activity

**Figure 3:**
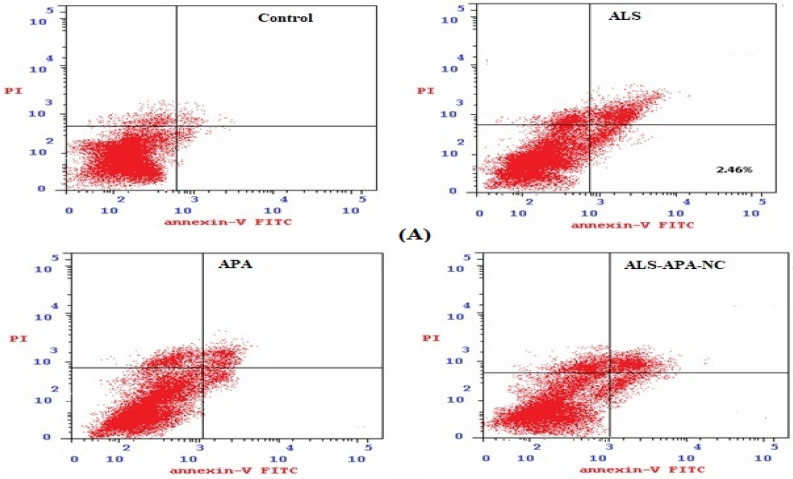

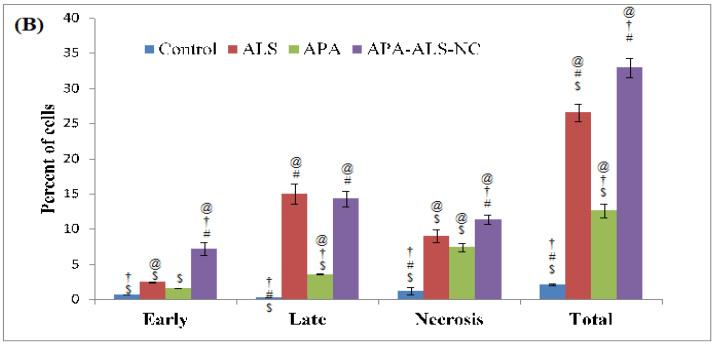
(**A**) Flow cytometry data showing the distribution of cells in control and treated PANC-1 cells. (**B**) Bar graphs represent apoptotic activity of control, ALS, APA, and APA-ALS-NC samples on PANC-1 cells. (Statistical inferences: ^@^ *p* < 0.05, in comparison to control; ^†^ *p* < 0.05, in comparison to ALS; ^#^ *p* < 0.05, in comparison to APA; and ^$^ *p* < 0.05, in comparison to APA-ALS-NC).

Apoptosis, an important cellular process, results in cytotoxicity. Thus, screening of apoptotic activity is essential in the development of anticancer agents. It is also useful in the evaluation of drug delivery systems. The results of apoptotic studies with the samples are provided in Figure 3. The results show that ALS results in a greater percentage of cells in the late phase but without a statistically significant difference (*p*-value > 0.05) with APA-ALS-NC. Meanwhile, APA-ALS-NC results in a significantly (*p*-value < 0.05) greater percentage of cells in all other phases. The results confirmed the higher necrotic effect of APA-ALS-NC compared to ALS and APA. The effect of APA was significantly less compared to ALS and APA-ALS-NC in terms of percentage of cells at all phases.

#### 2.2.3. Cell Cycle Analysis

**Figure 4:**
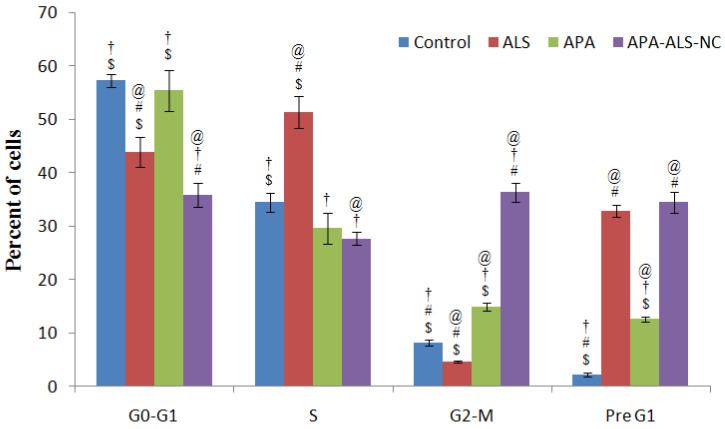
Cell cycle analysis results after treatment with control, ALS, APA, and APA-ALS-NC samples on PANC-1 cells. (Statistical inferences: ^@^ *p* < 0.05, in comparison to control; ^†^ *p* < 0.05, in comparison to ALS; ^#^ *p* < 0.05, in comparison to APA; ^$^ *p* < 0.05, in comparison to APA-ALS-NC).

The results of cell cycle analysis (Figure 4) demonstrated significant apoptotic activity of APA-ALS-NC. The higher percentage of cells in the G2-M and pre-G1 phases can be considered as an indication of the higher potential of the chemotherapeutic agent to induce apoptosis. In the case of the pre-G1 phase, the ALS and APA-ALS-NC samples showed a comparable percentage of cells. A similar apoptotic effect of ALS has been demonstrated in a previous study [38]. In the reported study, anti-proliferative and pro-apoptotic effects of ALS were evident. However, the percentage of cells in the G2-M phase was significantly higher (*p*-value < 0.05) for APA-ALS-NC. Thus, a significant increase in apoptotic activity was observed after the formulation of ALS to APA-ALS-NC.

#### 2.2.4. Mitochondrial Membrane Potential (MMP)

**Figure 5:**
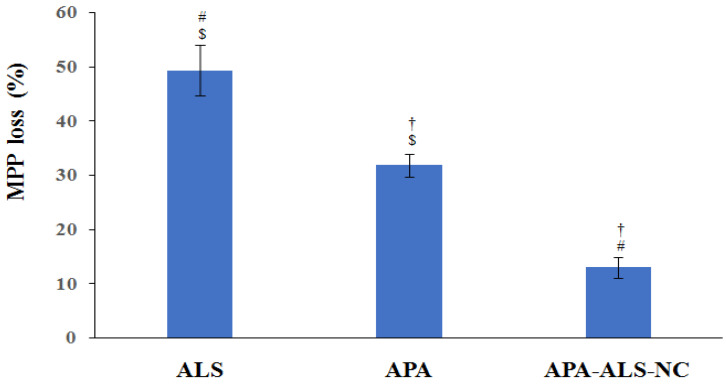
Mitochondrial membrane potential study results after treatment with ALS, APA, and APA-ALS-NC samples on PANC-1 cells. (Statistical inferences: ^†^ *p* < 0.05, in comparison to ALS; ^#^ *p* < 0.05, in comparison to APA; ^$^ *p* < 0.05, in comparison to APA-ALS-NC).

A change in MMP can occur, on exposure to apoptotic agents, due to the damage of the mitochondrial membrane [39]. This change can be measured for the purpose of comparison of cytotoxicity of samples. The results of the study of MMP after exposure to ALS, APA, and APA-ALS-NC samples are shown in Figure 5. Surprisingly, the results showed that APA-ALS-NC caused the smallest percentage loss in MMP. The highest percentage loss in MMP was caused by ALS. It has been demonstrated that bisphosphonates can inhibit mitochondrial adenine nucleotide translocase and subsequently influence MMP [40]. This might have contributed to the higher effect of ALS on MMP compared to other samples. APA also had a significant effect but less than that of ALS. Compounds such as APA have been reported to disrupt the mitochondrial membrane and therefore this observation can be justified [41]. However, the nanocomplexation of ALS and APA resulted in a lower influence on MMP.

#### 2.2.5. Determination of Marker Molecules by ELISA

**Figure 6:**
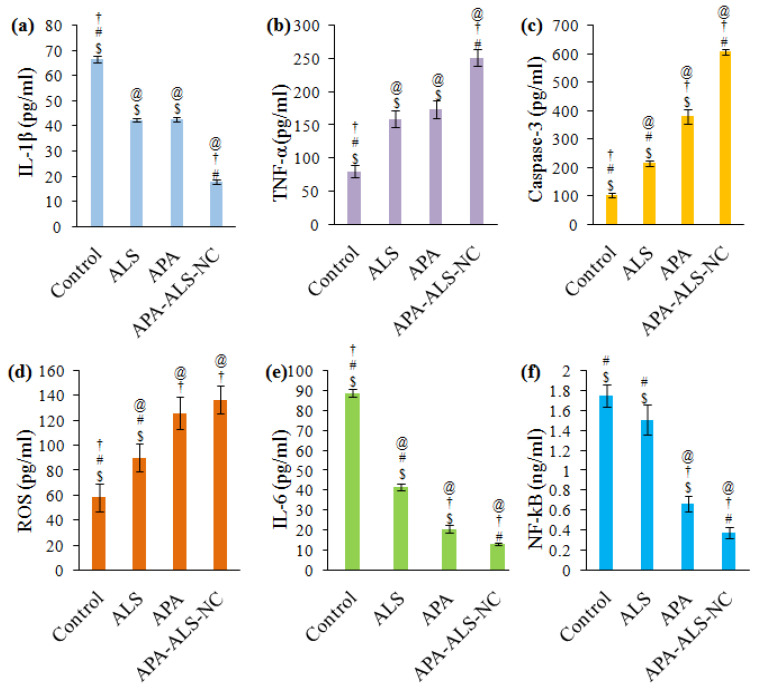
Effect of control, ALS, APA, and APA-ALS-NC samples on marker molecules (**a**) IL-1β, (**b**) TNF-α, (**c**) caspase-3, (**d**) ROS, (**e**) IL-6, (**f**) NF-kB. (Statistical inferences: ^@^ *p* < 0.05, in comparison to control; ^†^ *p* < 0.05, in comparison to ALS; ^#^ *p* < 0.05, in comparison to APA; ^$^ *p* < 0.05, in comparison to APA-ALS-NC).

The results of ELISA studies for the estimation of marker molecules are shown in Figure 6. These studies were carried out in order to ascertain the influence of the ALS, APA, and APA-ALS-NC samples in comparison to control on IL-1β, TNF-α, caspase-3, ROS, IL-6, and NF-kB.

IL-1β favors tumor growth and progression and thus a reduction in the concentration of IL-1β can be considered as the ability to suppress tumor growth and progression [42]. It was therefore inferred that APA-ALS-NC has significant (*p*-value < 0.05) tumor inhibition activity compared to all other samples (Figure 6a). The activities of APA and ALS were without any significant difference (*p*-value > 0.05). Thus, the formulation of ALS to APA-ALS-NC can be considered to enhance the suppression of tumor growth and progression by inhibiting IL-1β. TNF-α induces cytotoxicity and the production of higher levels of TNF-α can be considered as an indication of cytotoxicity of samples [43]. Thus, a significantly (*p*-value < 0.05) higher level of TNF-α produced by APA-ALS-NC can be attributed to its high cytotoxicity compared to ALS and APA samples (Figure 6b). Similar to that observed for IL-1β, the activities of APA and ALS on TNF-α were similar (*p*-value > 0.05). Increased levels of caspase-3 are indicative of higher cytotoxicity [44]. Thus, the significantly (*p*-value < 0.05) higher level of caspase-3 after APA-ALS-NC treatment compared to other samples indicated enhancement of cytotoxicity of ALS after formulation to APA-ALS-NC (Figure 6c).

In this study, total ROS was detected using antibodies. The ROS levels (Figure 6d) show that APA and APA-ALS-NC have comparable effects (*p*-value > 0.05). APA can augment ROS production and this might have contributed to the higher ROS levels on APA and APA-ALS-NC treatments [45]. However, their ROS levels were significantly (*p*-value < 0.05) higher than those produced by ALS. Meanwhile, the levels of IL-6 were significantly (*p*-value < 0.05) different for all the samples and followed the order control > ALS > APA > APA-ALS-NC (Figure 6e). Low levels of IL-6 increase the cytotoxicity of TNF-α [46]. Thus, samples that can reduce the expression of IL-6 can provide a higher cytotoxic effect. Therefore, APA-ALS-NC with the lowest expression of IL-6 could be expected to have higher toxicity than the other samples. In the case of NF-kB, low levels inhibit tumor growth and progression. Thus, samples that reduce NF-kB level favors higher cytotoxicity. The lowest level for NF-kB was observed for APA-ALS-NC (Figure 6f). This reduction in NF-kB level was statistically significant (*p*-value < 0.05) compared to all other samples. The ability of APA to reduce the expression of NF-kB is already reported [47], but the nanocomplex was able to reduce the level of NF-kB more significantly than APA.

#### 2.2.6. Estimation of Bax, Bcl-2, and p53 Gene Expressions Using RT-PCR

**Figure 7:**
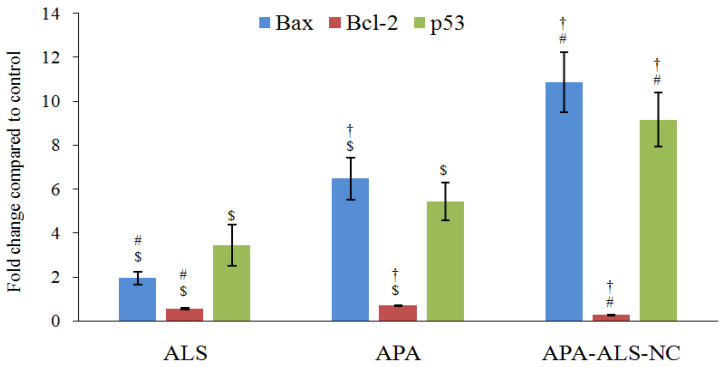
Effect of ALS, APA, and APA-ALS-NC samples on mRNA expression of Bax, Bcl-2, and p53. (Statistical inferences: ^†^ *p* < 0.05, in comparison to ALS; ^#^ *p* < 0.05, in comparison to APA; ^$^ *p* < 0.05, in comparison to APA-ALS-NC).

Determination of Bax and Bcl-2 gene expressions are useful in monitoring of apoptotic ability of samples. High levels of Bax indicate apoptosis whereas low levels of Bcl-2 favor apoptosis [48]. Bax expression was significantly higher (*p*-value < 0.05) for APA-ALS-NC compared to other samples (Figure 7). The Bax expression produced by APA was more than that produced by ALS. Nevertheless, a previous study reported a reduction in Bax expression by APA [49]. However, such an influence was not observed in the present study. In the case of Bcl-2 expression, APA-ALS-NC produced the lowest level corresponding to the highest apoptosis (Figure 7). The molecular structure of APA is favorable for the activation of p53. Thus, a higher effect of APA on p53 expression was expected. However, the Bcl-2 expression was found to be more for APA than ALS. Such an enhancement of Bcl-2 expression by APA is already reported [49]. p53 transcription factor produces apoptosis and higher expression can be considered favorable for apoptosis and cytotoxicity [50]. The effect on p53 expression was in the order APA-ALS-NC > APA > ALS (Figure 7). However, there was no significant difference (*p*-value < 0.05) in the p53 expression of samples treated with ALS and APA. In addition, APA-ALS-NC showed the highest cytotoxicity potential. Thus, the results of Bax, Bcl-2, and p-53 expressions confirmed that the formulation of ALS to APA-ALS-NC using APA significantly enhanced the cytotoxicity.

## 3. Materials and methods

### 3.1. Materials

Apamin (APA) was purchased from Sigma Aldrich (St. Louis, MO, USA), ALS was gifted from SPIMACO from Riyadh, Saudi Arabia. All other chemicals used in the study were of analytical reagent grade. The pancreatic cancer cell line (PANC-1) was procured using a cell strain from the American Type Cultural Collection (ATCC). PANC-1 cells were cultured in a DMEM medium supplemented with 10% fetal bovine serum (FBS), penicillin, and streptomycin (Gibco, Thermo Fisher Scientific, Grand Island, NY, USA).

### 3.2. Formulation and Optimization of APA-ALS Nanocomplex (APA-ALS-NC)

ALS-APA nanocomplex formulation was prepared according to the Box-Behnken design (Table 1). The design was generated and evaluated using Statgraphics software (Statgraphics Technologies, Inc., The Plains, VA, USA). ALS concentration (mM, X1), incubation time (min, X2), and sonication time (min, X3) were considered as independent variables whereas particle size was considered as a dependent variable. The numerical optimization of APA-ALS-NC was done by setting a minimum value for particle size as the goal. The optimized APA-ALS-NC was prepared using optimized formula having ALS (1.00171 mM) and APA (1 mM), placed in 20 mL of 0.01 M phosphate buffer 7.4 pH levels before being whirled for 2 min for dissolution. The incubation and sonication time were optimized as 10 min and 5 min, respectively.

#### Determination of Particle Size, Size Distribution, and Zeta Potential

The particle size, size distribution, and zeta potential of APA-ALS-NC were determined after 10 times dilution of the mixed solutions in the same buffer using Zetasizer Nano ZSP (Nano ZSP, Malvern, Worcestershire, UK). Transmission electron microscopy (TEM) analysis was performed and examined under TEM (JEOL JEM-HR-2100, JEOL, Ltd., Tokyo, Japan).

### 3.3. In Vitro Cell Line Studies of APA-ALS-NC in Pancreatic Cancer Cells (PANC-1 Cells)

#### 3.3.1. IC_50_ Determination Using MTT Assay

The IC_50_ determination was done in PANC-1 cell lines by MMT assay. The cells in 96-well plates (5 × 10^3^ cells/well) were permitted to attach by overnight incubation. The cells were subjected to treatment with APA-ALS-NC at different concentrations for 4 h at 37 °C. The supernatant was then removed and 100 μL of DMSO was used to solubilize the formazan formed after the sample treatment. The sample absorbance at 570 nm was determined in a microplate reader. In addition to the sample with ALS, APA and control (without any treatment) treatments were also carried out. The IC_50_ values were then determined (*n* = 3) and reported.

#### 3.3.2. Apoptotic Activity

The study was carried out following a previously reported method in PANC-1 cells [51]. The cells were subjected to incubation for 24 h with the IC_50_ concentration of formulation samples (control, ALS, APA, and APA-ALS-NC) in a 6-well plate (1 × 10^5^ cells/well). After sample treatment, the PANC-1 cells were centrifuged and separated. Thereafter, the cells were washed using phosphate-buffered saline and later re-suspended in 500 μL of 1X binding buffer. The staining of the PANC-1 cells was done using a commercially available kit (Annexin v-FITC/PI kit, K101-100, Biovision Inc, Milpitas, CA, USA) and following the prescribed procedure. The cells were quantified by flow cytometry (FACS Calibur, BD Bioscience, San Jose, CA, USA) and reported.

#### 3.3.3. Cell Cycle Analysis

Cell cycle analysis was carried out by flow cytometry. The procedure described for apoptotic activity was used for the cell cycle analysis too.

#### 3.3.4. Mitochondrial Membrane Potential (MMP)

MMP was determined by employing an assay kit with tetramethylrhodamine methyl ester (TMRM) as the probe. The cells at a density of 1.5 × 10^4^ cells/well were obtained in a 96-well plate and after 24 h, they were incubated with samples (ALS, APA, and APA-ALS-NC) in 300 μL DMEM medium (supplemented with 10% FBS and 1% antibiotics). After sample treatment, the medium was replaced with the probe solution and incubated in dark. After replacing the probe solution, the live-cell imaging buffer was added and flow cytometry was carried out [52].

#### 3.3.5. Determination of Marker Molecules by ELISA

Interleukin-1β (IL-1β), tumor necrosis factor-alpha (TNF-α), caspase-3, reactive oxygen species (ROS), interleukin-6 (IL-6), and nuclear factor kappa B (NF-kB) were the marker molecules studied. The analysis of molecular markers was done by ELISA kit for the biomarker. Briefly, PANC-1 cells (5 × 10^4^ cells/well in a 96-well plate) were treated with samples (ALS, APA, and APA-ALS-NC) and allowed to undergo equilibration at ambient temperature. Later, the reagent (100 μL) was added to each well containing 100 μL of the medium. After mixing for 30 s at 500 rpm, the samples were kept aside at ambient temperature for 30 min. Finally, IL-1β (R&D Systems, Inc. Minneapolis, MN, USA), TNF-α (Abcam, Waltham, MA, USA), caspase-3 (Invitrogen Corporation, Camarillo, CA, USA), ROS kit (ROS ELISA kit, Amsbio, Abingdon, UK), IL-6 (R&D Systems, Inc. Minneapolis, MN, USA), and NF-kB (MyBioSource, Inc, San Diego, CA, USA) were estimated using the corresponding ELISA kit.

#### 3.3.6. Estimation of Bax, Bcl-2, and p53 Gene Expressions Using RT-PCR

Bax, Bcl-2, and P53 gene expressions (mRNA) were analyzed in all samples by RT-PCR [53]. The Qiagen RNA extraction/BioRadsyber green PCR MMX kit was used in the study. A Rotorgene RT- PCR system was used for reading. The system was equipped with Rotor-Gene 1.7.87 software. The sequences of the primers used for the study are shown in Table 4.

### 3.4. Statistical Analysis

Statistical analysis was carried out to determine statistical significance by one-way ANOVA followed by Tukey’s multiple comparison test and *p*-value less than 0.05 (*p* < 0.05) was considered significant.

## 4. Conclusions

The nanocomplex using APA and ALS was prepared and optimized for particle size using a Box-Behnken design. The optimum formula contained 1.00171 mg ALS. The incubation time was 10 min and sonication time was 5.99857 min. The optimized APA-ALS-NC had a particle size of 161.52 nm. Various in vitro cell line studies of APA-ALS-NC were carried out in PANC-1 cells. The IC_50_ value of APA-ALS-NC was significantly lower compared to that of ALS and APA samples. Furthermore, the apoptosis studies revealed a higher necrotic effect of APA-ALS-NC compared to ALS and APA. The cell cycle analysis confirmed the significant apoptotic activity of APA-ALS-NC. Nevertheless, the APA-ALS-NC sample had the lowest effect on change in MMP. ALS produced the highest change in MMP. The levels of biomarkers showed that APA-ALS-NC can significantly enhance apoptosis and cytotoxicity in PANC-1 cells. Overall, the study results revealed that the formulation of ALS to APA-ALS-NC using APA significantly enhances the cytotoxicity towards PANC-1 cells.

## Figures and Tables

**Table 1:** The responses obtained (observed and predicted values) for various APA-ALS-NC formulation trials.

Run	Independent Factors			Dependent Factor	
	Factor A: ALS Amount (mM)	Factor B: Incubation Time (min)	Factor C: Sonication Time (min)	Response 1: Mean Particle Size (nm)	
				Observed	Predicted
1	1	50	5	192	190.5
2	5.5	90	5	342	334.5
3	1	90	3	218	227.0
4	10	10	3	389	380.0
5	5.5	50	3	321	319.3
6	10	50	1	421	422.5
7	1	50	1	191	186.0
8	5.5	50	3	319	319.3
9	5.5	50	3	318	319.3
10	5.5	10	1	298	305.5
11	1	10	3	176	173.5
12	10	50	5	363	368.0
13	10	90	3	432	434.5
14	5.5	10	5	265	269.0
15	5.5	90	1	352	348.0

**Table 2:** ANOVA data for particle size of APA-ALS-NC formulations.

Source	Sum of Squares	Degrees of Freedom	Mean Square	F-Ratio	*p*-Value
A: ALS (mM)	85,698.0	1	85,698.0	1133.07	0.0000
B: Incubation time (min)	5832.0	1	5832.0	77.11	0.0003
C: Sonication time (min)	1250.0	1	1250.0	16.53	0.0097
AA	1338.78	1	1338.78	17.70	0.0084
AB	0.25	1	0.25	0.00	0.9564
AC	870.25	1	870.25	11.51	0.0194
BB	44.1603	1	44.1603	0.58	0.4793
BC	132.25	1	132.25	1.75	0.2433
CC	269.391	1	269.391	3.56	0.1178
Total error	378.167	5	75.6333	--	--
Total (corr.)	95,795.7	14	--	--	--

**Table 3:** Optimum formula for the APA-ALS-NC formulation.

Factor	Low	High	Optimum
ALS (mM)	1.0	10.0	1.00171
Incubation time (min)	10.0	90.0	10.0
Sonication time (min)	1.0	5.0	4.99857

**Table 4:** Sequences of the primers used in real-time polymerase chain reaction (RT-PCR).

Bax F	5′-TGGCAGCTGACATGTTTTCTGAC-3′
Bax R	5′-TCACCCAACCACCCTGGTCTT-3′
Bcl-2 F	5′-TCGCCCTGTGGATGACTGA-3′
Bcl-2 R	5′-CAGAGACAGCCAGGAGAAATCA-3′
p53 F	5′-GACGGTGACACGCTTCCCTGGATT-3′
P53 R	5′-GGGAACAAGAAGTGGAGAATGTCA-3′
GAPDH F	5′-AATGCATCCTGCACCACCAA-3′
GAPDH R	5′-GATGCCATATTCATTGTCATA-3′

## Data Availability

The data presented in this study are available in article.
